# Effects of Astragaloside IV on the SDF-1/CXCR4 Expression in Atherosclerosis of apoE^−/−^ Mice Induced by Hyperlipaemia

**DOI:** 10.1155/2015/385154

**Published:** 2015-05-17

**Authors:** Hewei Qin, Ping Liu, Shengchao Lin

**Affiliations:** ^1^Longhua Hospital Affiliated to Shanghai University of Traditional Chinese Medicine, 725 South Wanping Road, Shanghai 200032, China; ^2^East China University of Science and Technology, Shanghai 200237, China

## Abstract

Astragaloside IV (AsIV) is the major effective component extracted from the Chinese herb *Astragalus membranaceus,* which has been widely used to treat cardiovascular disease. Recent studies have shown that AsIV can potentially protect the arteries from atherosclerosis; however the mechanisms underneath are unknown. The aim of this study was to investigate the effects of AsIV on blood lipids, CD40-CD40L signal system, and SDF-1/CXCR4 biological axis in high-fat diet apoE^−/−^ mice and reveal the molecular mechanisms of AsIV against atherosclerosis. Here, we showed that AsIV alleviated the extent of atherosclerosis in aorta of apoE^−/−^ mice. And AsIV can significantly downregulate PAC-1, CD40L, and CXCR4 expression on platelet surface in blood of high-fat diet apoE^−/−^ mice. AsIV also can significantly downregulate mRNA and protein level of SDF-1 and CXCR4 in thoracic aorta. Consistent with aorta CXCR4 expression, CXCR4 in bone marrow-derived endothelial progenitor cell (EPC) was also reduced. Meanwhile biochemical analysis showed that AsIV could downregulate TG, TC, and LDL-C levels and upregulate HDL-C level in blood of high-fat diet apoE^−/−^ mice. We concluded that the protective effects of AsIV in atherosclerosis injury may be related to regulating blood lipids, CD40-CD40L system, and SDF-1/CXCR4 biological axis. SDF-1/CXCR4 biological axis is probably one of the main targets of intervening atherosclerosis.

## 1. Introduction

Huang qi (*Astragalus membranaceus*) is a traditional Chinese herbal medicine that has been widely used in stroke patients in China for decades; astragaloside IV (AsIV) is a major active component of* Astragalus membranaceus*; its molecular structure, pharmacokinetics, and pharmacological actions have been well studied. Studies showed that AsIV plays a potential role in protecting the heart from myocardial ischemia, which may interpret its beneficial effects to treat cardiovascular diseases in Chinese herb medicine [1]. The mechanism of its action may be related to reducing lipid peroxidation, improving energy metabolism and inhibiting production of free radicals, and upregulation of several KATP channel subunits and facilitation of KATP currents [2].

Studies have suggested that platelets play a vital role in inflammatory reactions. Resting platelets can be activated when they are exposed to stimulants, damaged vessel wall, or extravascular tissue, accompanied by change in platelet shape and ability of adhesion, as well as aggregation. Platelet activation is an ordered sequence of events which begins with the interaction between the adhesive protein (agonist) and its receptor; the activation of platelets leads to secretion of a variety of mediators which can lead to progress of atherosclerosis, such as cytokines, chemokines, growth factors, adhesion molecules, and coagulation factors [3–5].

There are markers that can be used for platelet activation, such as integrin *α*IIb*β*3 (PAC-1) and CD40 ligand (CD40L). CD40L is one of the receptors which translocate to the platelet surface upon platelet activation and constitutively expresses on platelets; CD40-CD40L interactions play a central role in immune responses and inflammation; PAC-1 is key part in platelet aggregation through interaction with fibrinogen. Also researchers reported that stromal cell-derived factor 1 (stromal cell-derived factor-1, SDF-1) was upregulated upon platelet activation; SDF-1 and its receptor CXCR4 (CXC chemokine receptor 4) composed SDF-1/CXCR4 axis which is related to the formation of atherosclerosis; expression of SDF-1 is enhanced on platelet surface in atherosclerosis plaques [6–8]. AMD3100 is an antagonist of CXCR4, which can disrupt binding of SDF-1 to CXCR4 by competing for the binding site, thus blocking the physiological function of SDF-1/CXCR4 axis. Moreover, SDF-1/CXCR4 axis has been shown to play critical roles in stem cell mobilization, migration, and homing and in immunoregulation, inflammatory disease, and autoimmune disorder [9, 10].

In the present study, we measured the level of biomarkers of platelet activation, PAC-1, CD40L, and CXCR4 in platelet-rich plasma, so as to investigate the effect of AsIV on platelet activation. We also examined the expression of SDF-1/CXCR4 biological axis in apoE^−/−^ mice. The effects of astragaloside IV on atherosclerosis in high-fat diet apoE^−/−^ mice may be through platelet activation and SDF-1/CXCR4 biological axis.

## 2. Materials and Methods

### 2.1. Animals

30 healthy male apoE^−/−^ mice at 8 weeks old were provided by Model Animal Research Center of Nanjing University, specific pathogen-free grade, weighing 20 ± 2 g. Another 12 healthy male inbred C57BL/6 mice at 8 weeks old were obtained from Shanghai SLAC Laboratory Animal Company, specific pathogen-free grade, weighing 20 ± 2 g. All animals were housed on a 12 : 12 light/dark cycle in the experimental animal center of Longhua Hospital Affiliated to Shanghai University of Traditional Chinese Medicine.

### 2.2. Drugs and Reagents

Astragaloside IV (purity > 98%, CAS: 84687-43-4) was purchased from Nanjing Spring & Autumn Biological Engineering Co. (Nanjing, China). AMD3100 octahydrochloride hydrate (A5602-5MG, P-code: 1001580646–1001580659) was from Sigma-Aldrich Co. LLC (St. Louis, MO, USA).

Immunohistochemical kit was purchased from Beijing Zhongshan Reagent Co. Ltd. Kit of SABC (SA2002) and kit of DAB chromogenic (AR1022) were purchased from Wuhan Boster Company (Wuhan, China).

### 2.3. Animal Model and Grouping

Mice in blank control group were given normal diet. All apoE^−/−^ mice were given high-fat western diet whole diet (21% fat, cholesterol, 0.15%) till 2 weeks old, three of them were chosen randomly to be sacrificed in order to observe the success of modeling. apoE^−/−^ mice were randomly divided into three groups: model group, AMD3100 groups, and AsIV group; C57BL/6 mice were used as the control group.

Mice in AsIV group were given 40 mg/kg·d^−1^ astragaloside by oral gavage. Mice in AMD3100 group were given 2.5 mg/kg·2d^−1^ AMD3100 by intraperitoneal injection. Mice of model group and the normal group were given 0.9% sodium chloride solution by oral gavage. Mice of each group continued to be fed following the original feeding, within all 12 weeks.

### 2.4. Collection of Venous Blood and Aorta

After 12 weeks of drug administration, mice were fasted for 12 hours, and venous blood was collected through the eyeball under sterile condition. Then open the chest and abdominal cavity quickly, and peel total length of the aorta along the aorta valve to the iliac artery branch.

### 2.5. Detection of Blood Lipid Index

After 30-minute standing, venous blood was obtained and centrifuged at 3000 rpm for 15 min. Serum was isolated. Total cholesterol [11], triacylglycerol (TG), high-density lipoprotein (HDL-C), and low-density lipoprotein (LDL-C) levels were detected by automatic biochemical analyzer. Kit of total cholesterol, Kit of TC and TG was purchased from Wenzhou Dongou Ma Jin Biotech Co., Ltd (Wenzhou, China). Kit of HDL-C and LDL-C was purchased from Beijing North of Conde Clinical Reagent Co. (Beijing, China). Kit of apolipoprotein A I (ApoA I) and apolipoprotein B (ApoB) was purchased from Shanghai Rongsheng Pharmaceutical Co. (Shanghai, China).

### 2.6. Histological Analyses

Thoracic aorta was isolated, fixed, and embedded in paraffin for histopathological analysis. Hematoxylin and eosin (H&E) staining was performed. Imaging from aorta tissues was detected with microscope. Detection using Image Pro Plus: luminal area (LA), intimal thickness (IMT), plaque area (PA), fibromuscular component (FS), a lipid center area (CA), and minimum fibrous cap thickness (FCT).

### 2.7. Detection of Platelet Activation

Platelet-rich plasma was prepared from flesh blood of the mice by density gradient centrifugation as described by Hoffman's report [12]. Each sample was then analyzed by FACScan (BD Bioscience) to detect PAC-1, CD40L, and CXCR4 level on platelet surface. FITC-conjugated PAC-1 antibody, PE-conjugated CD40L antibody, and APC-conjugated CXCR4 antibody were all purchased from GeneTex Co.

### 2.8. Immunohistochemical Stain

Immunohistochemical staining was performed as described [13] with the following antibodies: anti-SDF-1 (GeneTex, GTX45117) and anti-CXCR4 (GeneTex, GTX53457). Antigen granular or diffuse coloring, tan or brown pigmented cells are regarded as positive cells. The results analyzed using Image-Pro Plus image analysis system. In each section, five positive coloring regions were randomly selected and the average optical density of cells was determined.

### 2.9. Isolation and Culture of Bone Marrow-Derived Endothelial Progenitor Cells (EPC)

Bone marrow-derived EPC were isolated from femurs of apoE^−/−^ mouse bone marrow (Bm) progenitor cells and cultured in endothelial basal medium-2 (EBM-2) supplemented with growth factors (EBM-2; Lonza, catalogue number CC-3156) and 10% FCS as previously reported [14]. All experiments were performed with day 10 EPC cultures.

### 2.10. RNA Extraction and Quantification

Total RNA of bone marrow-derived endothelial progenitor cells and aorta tissue were extracted with the TRIzol reagent (Invitrogen Corporation, California, USA) reverse transcription system (TaKaRa Biotechnology Ltd., Shandong, China). Real-time PCR was performed using SYBR Green SuperMix with an iCycler thermal cycler (Bio-Rad Laboratories Inc., California, USA). Primer sequences of SDF-1 and CXCR4 are listed (SDF-1: F: 5′-CCTGTGTGTCATGCCCTCTT-3′ and R: 5′-AGTCCAGCCTGCTATCCTCA-3′; CXCR4: F: 5′-GTCAACCTCTACAGCAGCGT-3′ and R: 5′-CTATCGGGGTAAAGGCGGTC-3′). The data were collected and analyzed using the comparative Ct (threshold cycle) method. GADPH RNA was used as internal control.

### 2.11. Western Blot Analysis

Proteins of bone marrow-derived endothelial progenitor cells and aorta tissue were extracted and separated in SDS-PAGE gels, and western blot analyses were performed according to standard procedures as previously described [15]. Protein concentrations were determined using the BCA assay (Pierce Biotechnology Inc., Rockford, USA). Equal amounts of proteins (30 *μ*g) were prepared for western blotting assay. *β*-Actin was used as loading controls. The primary antibodies against SDF-1 and CXCR4 were all purchased from GeneTex Co.

### 2.12. Statistical Method

Data were denoted by mean value ± standard deviation ($\bar{x}\pm s$). Variance analysis was adopted. Comparison between two groups was carried out by SNK method. *P* < 0.05 indicated significant differences; and statistical calculation was accomplished by SPSS18.0 software.

## 3. Results

### 3.1. Effects of AsIV on Animal Blood Lipid

The results of TC, TG, HDL-C, and LDL-C levels of mouse in model group, AMD3100 groups, and AsIV group were shown in Table 1. Serum TC, LDL-C, and TG levels were increased and HDL-C was reduced more significantly in model group, compared with control group (all *P* < 0.01). In AsIV group, levels of TC, TG, and LDL-C were lower than those in model group and AMD3100 group (all *P* < 0.05), while level of HDL-C in AsIV group was significantly higher than that in model group and AMD3100 group (*P* < 0.05).

### 3.2. Histopathological Assessment

**Figure 1:**
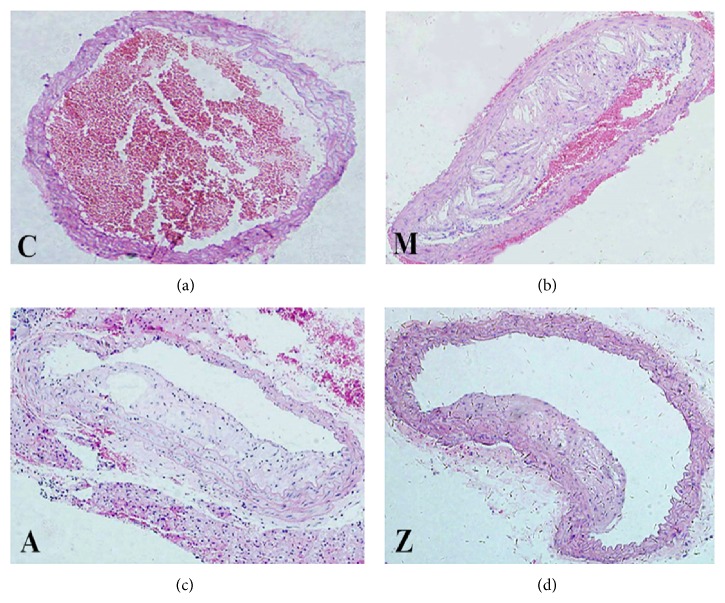
Hematoxylin and eosin stained histological sections. Note: C: control group; M: model group; A: AMD3100 group; Z: AsIV group (magnification ×200).

To assess the extent of atherosclerosis in thoracic aorta of high-fat diet apoE^−/−^ mice after AMD3100 or AsIV treatment, aorta cross-section pathological damage was detected by HE staining. Histopathological specific data analysis (Tables 2, 3, and 4) suggested that, compared with the model group, aorta pathology of AsIV group and AMD3100 group showed that lumen areas (LA) were larger, intima medium thickness (IMT) was thinner, plaque area (PA) was smaller, fiber structure (FS) was smaller, cholesterol area (CA) was smaller, fiber cap thickness (FCT) was thinner, PA/LA was smaller, CA/PA was larger, and CA/FS was larger, with all data showing significant differences (*P* < 0.05). Compared with the AMD3100 group, aorta pathology of AsIV group showed that LA was larger, IMT was thinner, PA was smaller, FS was smaller, CA was smaller, FCT was thinner, PA/LA was smaller, CA/PA was larger, and CA/FS was larger, with all data showing significant differences (*P* < 0.05). Examples of each group were showed in Figure 1.

### 3.3. Effects of AsIV on PAC-1, CD40L, and CXCR4 Expression of Platelet Surface

**Figure 2:**
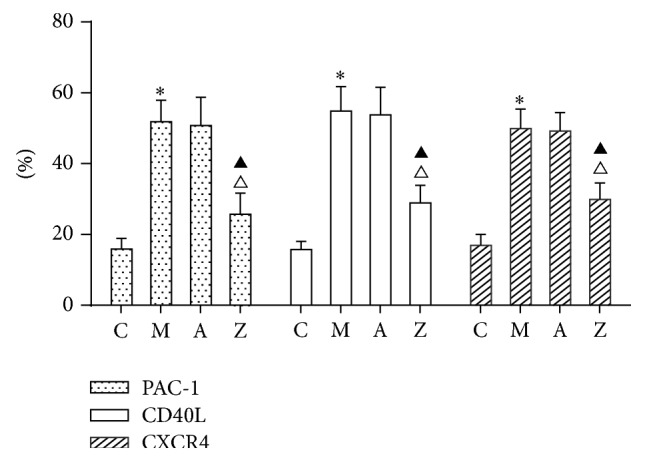
PAC-1, CD40L, and CXCR4 expression of platelet surface. Note: C: control group; M: model group; A: AMD3100 group; Z: AsIV group. Compared with control group: ^∗^ *P* < 0.05; compared with model group: ^△^ *P* < 0.05; compared with AMD3100 group: ^▲^ *P* < 0.05.

To investigate the effect of AsIV on the activation of platelet, biomarkers of platelet activation were measured by flow cytometry. Results showed that expression of PAC-1, CD40L, and CXCR4 was significantly higher in the model group than in the control group (*P* < 0.05). Compared with the model group, the expression of PAC-1, CD40L, and CXCR4 was significantly decreased in AsIV group (*P* < 0.05). Compared with the AMD3100 group, the expression of PAC-1, CD40L, and CXCR4 was significantly decreased in AsIV group (*P* < 0.05).  Figure 2 showed the results.

### 3.4. Effects of AsIV on SDF-1 and CXCR4 Levels in Mice Aorta Wall

**Figure 3:**
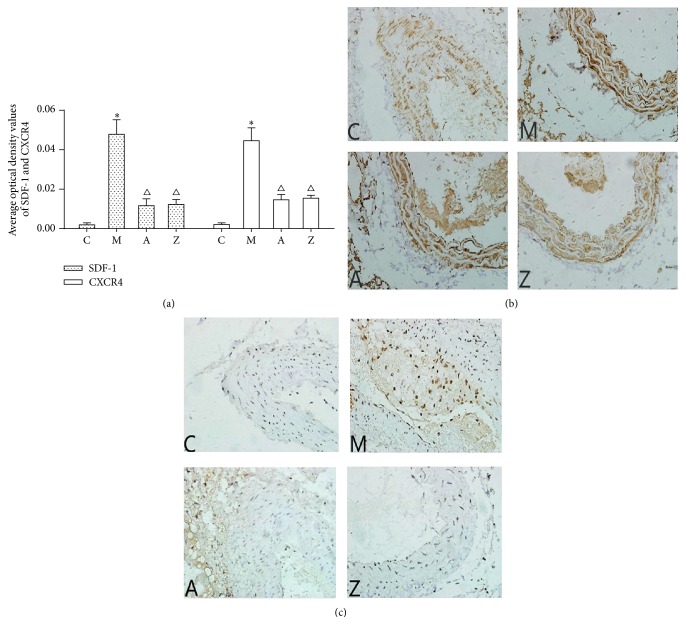
(a) The expression of SDF-1 and CXCR4 (optical density value). Compared with control group: ^∗^ *P* < 0.05; compared with model group: ^△^ *P* < 0.05. (b) Expression of SDF-1 (magnification ×400). (c) Expression of CXCR4 (magnification ×400). Note: C: control group; M: model group; A: AMD3100 group; Z: AsIV group.

Immunohistochemical staining was applied to investigate the effect of AsIV on SDF-1/CXCR4 biological axis in aorta wall of the high-fat diet apoE^−/−^ mice.  Figure 3(a) illustrated that expression of SDF-1 and CXCR4 in model group was significantly higher than that of control group (*P* < 0.05). However, in AMD3100 group and AsIV group, SDF-1 and CXCR4 had lower expression in the aorta smooth muscle cells than in model group. Compared with the AMD3100 group, average optical density values of SDF-1 and CXCR4 in AsIV group were higher, but the difference was not statistically significant (*P* > 0.05). Examples of each group were showed (Figures 3(b) and 3(c)).

### 3.5. Effects of AsIV on Expression of SDF-1 and CXCR4 in mRNA Level and Protein Level

**Figure 4:**
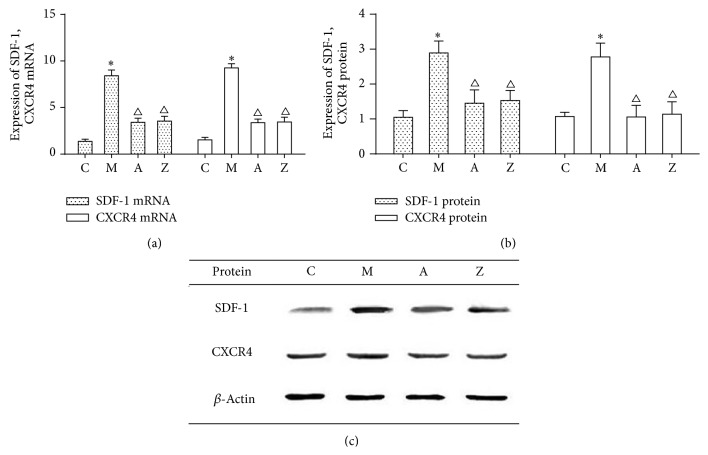
(a) The expression of SDF-1, CXCR4 mRNA. (b, c) The expression of SDF-1, CXCR4 protein. Note: C: control group; M: model group; A: AMD3100 group; Z: AsIV group. Compared with control group: ^∗^ *P* < 0.05; compared with model group: ^△^ *P* < 0.05.

We further examined the expression of SDF-1 and CXCR4 in mRNA level and protein level in aorta by quantitative PCR and western blotting analysis. Quantitative real-time PCR demonstrated that mRNA level of SDF-1 and CXCR4 was significantly lower in AMD3100 group and AsIV group than in model group (Figure 4(a)). However, compared with the AMD3100 group, the mRNA levels of SDF-1 and CXCR4 were higher in the AsIV group, but the difference was not statistically significant (*P* > 0.05).

Moreover, western blotting demonstrated that proteins level of SDF-1 and CXCR4 was significantly lower in AMD3100 group and AsIV group than in model group. However, compared with the AMD3100 group, the proteins levels of SDF-1 and CXCR4 were higher in the AsIV group, but the difference was not statistically significant (*P* > 0.05) (Figures 4(b) and 4(c)). The expression of SDF-1 and CXCR4 proteins showed highly consistence with the mRNA level after AMD3100 and AsIV treatment.

### 3.6. Effects of AsIV on CXCR4 Expression in Bone Marrow-Derived EPC from apoE^−/−^ Mice

**Figure 5:**
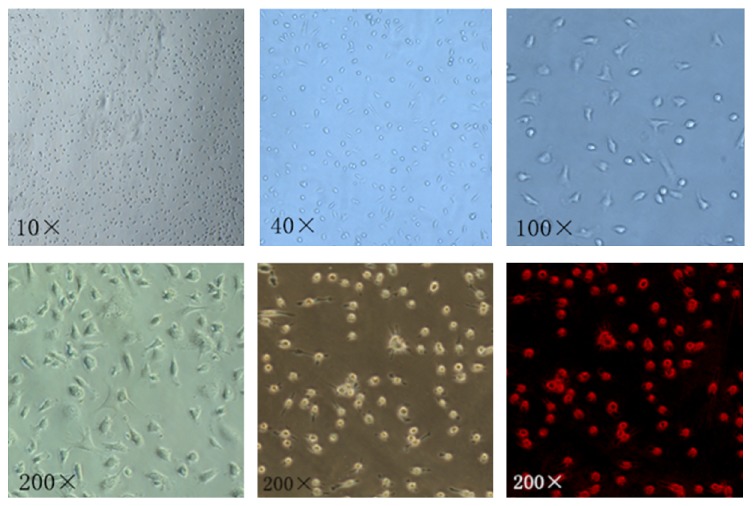
Identification of bone marrow-derived EPC (magnification ×200, VEGF-R positive cells, white shot; PE labeled VEGF-R antibodies stimulate green shoot).

**Figure 6:**
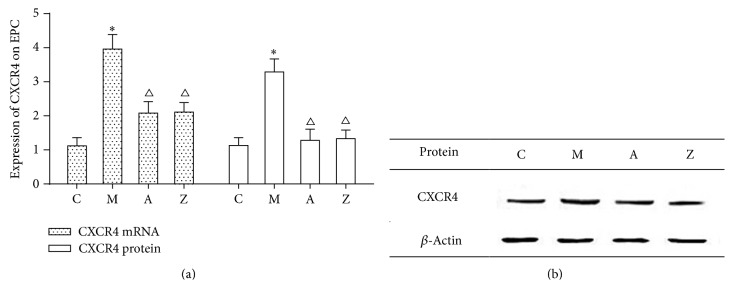
The expression CXCR4 on EPC. Note: C: control group; M: model group; A: AMD3100 group; Z: AsIV group. Compared with control group: ^∗^ *P* < 0.05; compared with model group: ^△^ *P* < 0.05.

To further verify the effect of AsIV SDF-1/CXCR4 biological axis associated with atherosclerosis, bone marrow-derived EPC were isolated and cultured (Figure 5). CXCR4 mRNA and protein levels in bone marrow-derived EPC were then detected. Quantitative real-time PCR and western blot demonstrated that the expression of CXCR4 mRNA and protein was significantly higher in the model group than in the control group (*P* < 0.05). Compared with the model group, the expression of CXCR4 mRNA and protein was significantly decreased in the AMD3100 group and AsIV group (*P* < 0.05). Compared with the AMD3100 group, the expression of CXCR4 mRNA and protein in AsIV group was higher, but the difference was not statistically significant (*P* > 0.05) (Figures 6(a) and 6(b)).

## 4. Discussion

The pathology of atherosclerosis development is a comprehensive long-term process. Due to its complex pathogenesis, there is no existing preventive and control strategy. “Injury-response theory” and the “inflammatory reaction theory” are the most popular explanation theories [16].

Targeting the key component in the atherosclerosis inflammatory response network can interrupt the formation of atherosclerosis and reduce the degree of injury. Recently, an increasing number of reports show that Chinese medicine has extraordinary effects on the treatment of atherosclerosis, which gradually draw people's attention to Chinese medicine. Huang qi (*Astragalus membranaceus*) is described as the dried root of leguminous plants in the Chinese Pharmacopoeia, it can help regeneration, and it also has effect on cardiovascular disorders, hepatitis, kidney disease, and skin diseases. Astragaloside IV is a major active component of the native* Astragalus membranaceus*. During decade, several researches have focused on studying extraction separation, pharmacokinetics, and pharmacological activities of astragaloside IV by separation and pure. It has been reported that astragaloside IV can promote zebrafish* in vivo*, which is closely associated with increase in the expression of vascular endothelial cell growth factor and its receptor, thereby activating the pathway of protein kinase B and phosphoinositide 3-kinase, as well as regulation of the expression of hypoxia inducible factor protein [17]. With regard to protection of endothelial function, researches showed that astragaloside IV can resist lipoprotein-induced injury to endothelial cells and increase the level of malondialdehyde (MDA) and SOD [18].

Recently, the role of CD40/CD40 ligand (CD40L) interactions in atherothrombosis, in the response of the immune system to pathogens and in thrombosis is now widely accepted. CD40-CD40L interactions have been identified in atherosclerosis, and such interactions can destabilize atherosclerotic plaques by inducing the expression of cytokines, chemokines, growth factors, matrix metalloproteinases, and procoagulant factors. Many literatures report that activated platelets can lead to overexpression of SDF-1; moreover, conglutination between SDF-1 and chemokine receptor CXCR4 can regulate cell migration, tissue targeting, and homing [19]. Based on the above evidence, our assumption is that astragaloside IV can target CD40-CD40L and therefore intervenes in atherosclerosis.

A latest study shows that atherosclerosis was related to SDF-1/CXCR4 biological axis. SDF-1, also known as CXCL12, belonged to CXC subfamily of chemokines. It was first discovered in cytokines secreted by mouse bone marrow stromal cells. After platelet activation, SDF-1 can be abundantly expressed and combined with chemokine receptors, CXCR4. SDF-1/CXCR4 biological axis consisting of SDF-1 and its receptor CXCR4 induced CD34+ stem cells to differentiate into macrophages and foam cells, which later caused the atherosclerosis. CXCR4 is widely expressed on surfaces of hematopoietic stem cells and bone marrow stromal cells; it is also expressed in AS lesions. Interaction of SDF-1 and CXCR4 control cell migration and issue-selective homing, which cause blood cells adhere to endothelial cells more conducive. Treating endothelium with chemokines suggests that SDF-1/CXCR4 axis plays an important role in the development of atherosclerosis [20]. On the other hand, SDF-1/CXCR4 biological axis is associated with bone marrow-derived endothelial progenitor cells during the development of atherosclerosis. Later on, Asahara et al. isolated and cultured progenitor cells from peripheral blood progenitor cells [21]. Werner et al. found that endothelial progenitor cells have independent predictive value for atherosclerotic disease [22]. On the basis of all these studies, we strongly assume that SDF-1/CXCR4 biological axis is targeted by astragaloside IV which explains how it intervenes in atherosclerosis.

apoE knockout (apoE^−/−^) mice were used as the animal model in our study. apoE^−/−^ mice fed by high-fat diet had the lesion characteristics of serious dyslipidemia and atherosclerosis, which were relatively good animal model for the research on atherosclerosis. In this research, C57BL mice had the characteristics of short life time and generation time, which were a kind of experimental animal commonly used in geriatric medicine.

In our research, we observed the effects of AsIV on blood lipids, CD40-CD40L signal system, and SDF-1/CXCR4 biological axis in high-fat diet apoE^−/−^ mice; the research results showed that TG, TC, HDL-C, and LDL-C levels in AsIV group were significantly better than those in model group (*P* < 0.05), AsIV significantly downregulated PAC-1, CD40L, and CXCR4 expression of platelet surface in blood of high-fat diet apoE^−/−^ mice. The extent of atherosclerosis in aorta of apoE^−/−^ mice in AsIV group was significantly lighter than that in model group, and the SDF-1 and CXCR4 expression of aorta reduced, showing statistical significance (*P* < 0.05). Western blotting and real-time PCR demonstrated that astragaloside IV significantly downregulated protein and mRNA expression of SDF-1 and CXCR4 (*P* < 0.05 versus model group), showing statistical significance. Consistent with this, astragaloside IV significantly downregulated protein and mRNA expression of CXCR4 of bone marrow-derived endothelial progenitor cell (*P* < 0.05 versus model group).

## 5. Conclusions

By above results, we can clearly know that platelet activation can induce SDF-1/CXCR4 biological axis imbalance. The protective effects of AsIV in atherosclerosis injury may be related to AsIV downregulation of CD40L, PAC-1, and CXCR4 expression by blocking the CD40-CD40L system. In addition, AsIV significantly down-regulated mRNA and protein level of SDF-1, CXCR4 in thoracic aorta. SDF-1/CXCR4 biological axis is probably one of the main targets of intervening atherosclerosis. Therefore, astragaloside IV plays a role in atherosclerosis of high-fat diet apoE^−/−^ mice by regulating blood lipids, CD40-CD40L system, and SDF-1/CXCR4 biological axis probably. The research provides new approach for treatment of atherosclerosis and related diseases.

## Figures and Tables

**Table 1:** Comparison of blood lipid (mmol/L, $\bar{x}$ ± *s*, *n* = 10).

Group	TC	TAG	LDL-C	HDL-C
Control group	4.16 ± 1.59	1.47 ± 0.18	1.11 ± 0.23	2.59 ± 0.12
Model group	16.12 ± 0.95^∗^	12.75 ± 2.65^∗^	6.48 ± 0.81^∗^	1.12 ± 0.13^∗^
AMD3100 group	16.10 ± 0.93	12.64 ± 2.25	6.28 ± 0.91	1.13 ± 0.13
AsIV group	10.96 ± 1.32^△▲^	4.78 ± 0.86^△▲^	4.30 ± 0.76^△▲^	2.90 ± 0.39^△▲^

Note: compared with control group: ^*^ *P* < 0.05; compared with model group: ^△^ *P* < 0.05; compared with AMD3100 group: ^▲^ *P* < 0.05.

**Table 2:** Comparison of LA, IMT, and PA ($\bar{x}$ ± *s*, *n* = 10).

Group	LA (mm^2^)	IMT (*μ*m)	PA (mm^2^)
Model group	0.255 ± 0.007	0.159 ± 0.047	0.158 ± 0.016
AMD3100 group	0.447 ± 0.042^△^	0.074 ± 0.013^△^	0.076 ± 0.018^△^
AsIV group	0.532 ± 0.017^△▲^	0.028 ± 0.011^△▲^	0.012 ± 0.013^△▲^

Note: compared with model group: ^△^ *P* < 0.05; compared with AMD3100 group: ^▲^ *P* < 0.05.

**Table 3:** Comparison of FS, CA, and FCT ($\bar{x}$ ± *s*, *n* = 10).

Group	FS (mm^2^)	CA (mm^2^)	FCT (*μ*m)
Model group	0.065 ± 0.002	0.093 ± 0.012	13.724 ± 0.443
AMD3100 group	0.031 ± 0.005^△^	0.041 ± 0.011^△^	4.136 ± 0.672^△^
AsIV group	0.002 ± 0.003^△▲^	0.013 ± 0.012^△▲^	2.306 ± 0.558^△▲^

Note: compared with model group: ^△^ *P* < 0.05; compared with AMD3100 group: ^▲^ *P* < 0.05.

**Table 4:** Comparison of PA/LA, CA/PA, and CA/FS ($\bar{x}$ ± *s*, *n* = 10).

Group	PA/LA	CA/PA	CA/FS
Model group	2.718 ± 0.386	0.581 ± 0.150	1.493 ± 0.290
AMD3100 group	0.690 ± 0.130^△^	0.776 ± 0.070^△^	2.516 ± 0.317^△^
AsIV group	0.041 ± 0.023^△▲^	0.797 ± 0.116^△▲^	3.546 ± 0.913^△▲^

Note: compared with model group: ^△^ *P* < 0.05; compared with AMD3100 group: ^▲^ *P* < 0.05.
